# Spinal Intramedullary Metastasis of Breast Cancer

**DOI:** 10.1155/2014/583282

**Published:** 2014-11-13

**Authors:** Recep Basaran, Mehmet Tiryaki, Dilek Yavuzer, Mustafa Efendioglu, Ece Balkuv, Aydin Sav

**Affiliations:** ^1^Dr. Lutfi Kirdar Kartal Education and Research Hospital, Department of Neurosurgery, Kartal, 34890 Istanbul, Turkey; ^2^Dr. Lutfi Kirdar Kartal Education and Research Hospital, Department of Pathology, 34890 Istanbul, Turkey; ^3^Istanbul Medeniyet University Goztepe Education and Research Hospital, Department of Neurology, 34890 Istanbul, Turkey; ^4^Acibadem University School of Medicine, Department of Pathology, 34890 Istanbul, Turkey

## Abstract

*Objective.* Breast cancer accounts for approximately one-third of all cancers in females. Approximately 8.5 % of all central nervous system metastases are located in the spinal cord. These patients have rapidly progressing neurological deficits and require immediate examination. The aim of surgery is decompression of neural tissue and histological evaluation of the tumor. In this paper, we present a case of breast cancer metastasis in thoracic spinal intramedullary area which had been partially excised and then given adjuvant radiotherapy. *Case.* A 43-year-old female patient with breast cancer for 8 years was admitted to our hospital with complaints of weakness in both legs. Eight years ago, she received chemotherapy and radiotherapy. On her neurological examination, she had paraparesis (left lower extremity: 2/5, right lower extremity: 3/5) and urinary incontinence. Spinal MRI revealed a gadolinium enhancing intramedullary lesion. Pathologic examination of the lesion was consistent with breast carcinoma metastasis. The patient has been taken into radiotherapy. *Conclusion.* Spinal intramedullary metastasis of breast cancer is an extremely rare situation, but it has a high morbidity and mortality rate. Microsurgical resection is necessary for preservation or amelioration of neurological state and also for increased life expectancy and quality.

## 1. Introduction

Breast cancer is the most common form of cancer in females in European countries. It accounts for one-third of all cancers in females [1]. Each year, approximately over 1 million women are diagnosed with breast cancer [2]. Distant metastasis is common [3]. Nearly 8.5% of all central nervous system metastases are into spinal cord, but the exact incidence of breast cancer's spinal intramedullary metastasis is not known [4]. It is predicted that only 0.1–0.4% of all cancer patients have metastasis into intramedullary spinal cord (MISC) [5]. These patients have rapidly progressing neurological deficit and require immediate evaluation. Spinal magnetic resonance imaging (MRI) is used for diagnosis. The aim of the surgery is the decompression of neural tissue and histological evaluation of the tumor. MISC has a very poor life expectancy like other central nervous system metastases. Mean survival time is about 3-4 months after diagnosis [6].

In this paper, we present a case of breast cancer metastasis in thoracic spinal intramedullary area which had been partially excised and then given adjuvant radiotherapy.

## 2. Case

**Figure 1:**
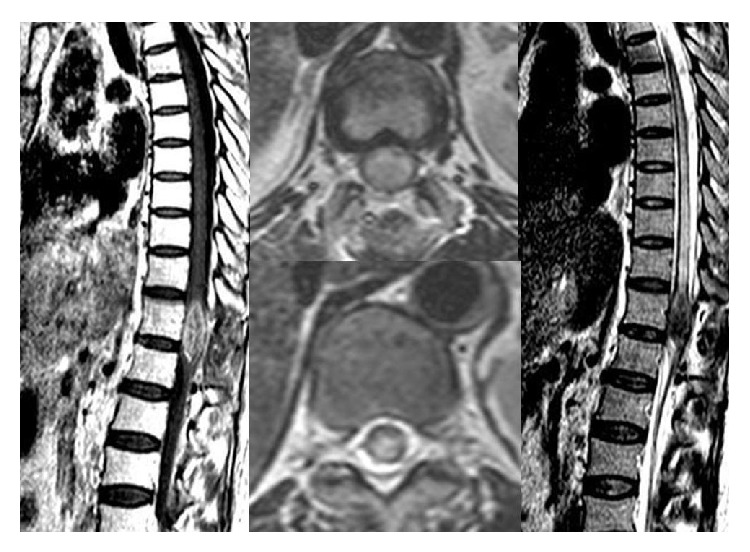
Spinal MRI showed an intramedullary and highly gadolinium enhancing lesion with regular borders.

**Figure 2:**
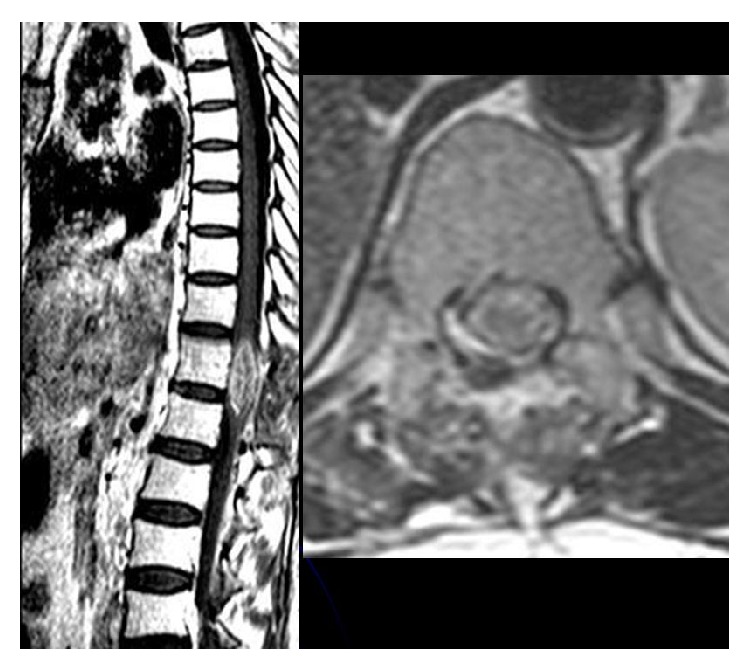
Postoperative spinal MRI showed not only evacuated inner part of the lesion but also remnant tumor adherent to neural tissue.

**Figure 3:**
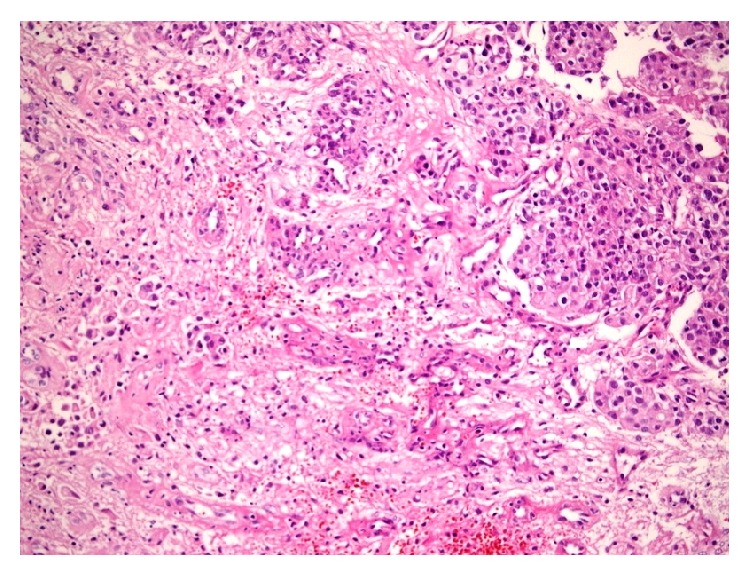
Epithelial tumour infiltration in neuroglial tissue (hematoxylin and eosin, ×200).

**Figure 4:**
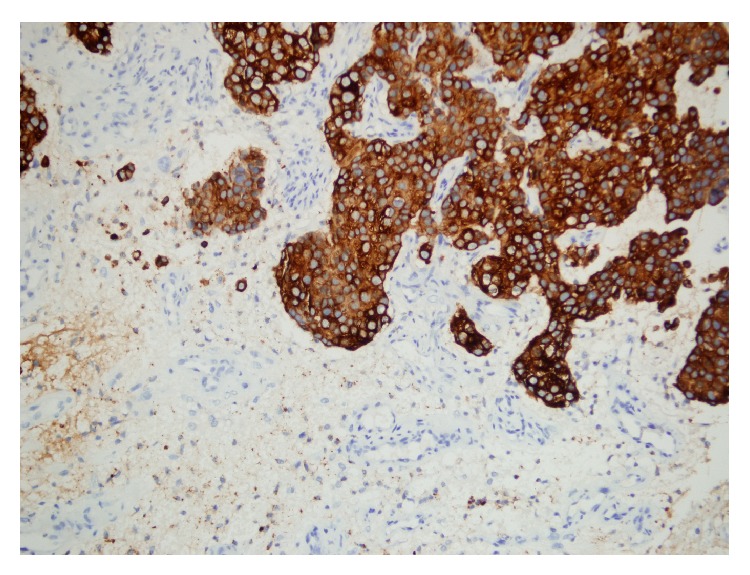
Cytokeratin expression in tumor tissue (pancytokeratin, ×200).

**Figure 5:**
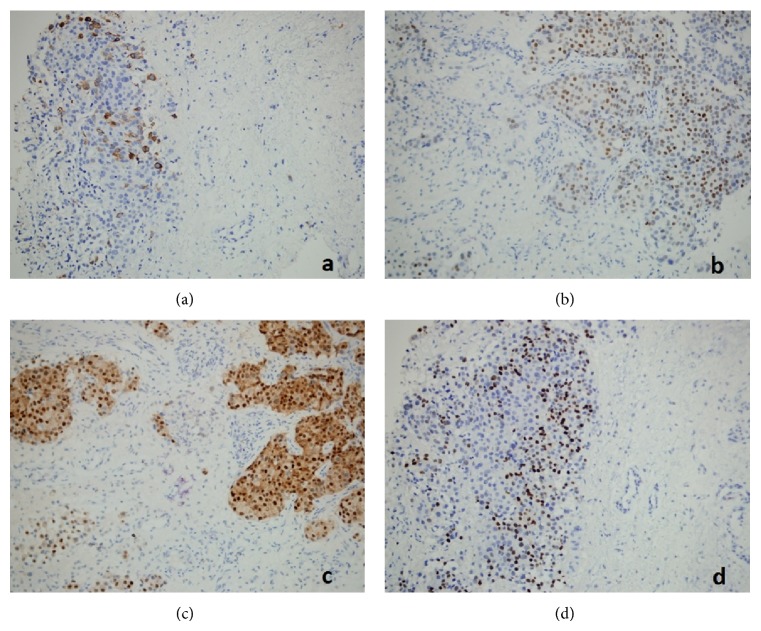
(a) Mammaglobin expression of tumor cells (×200), (b) estrogen receptor status of tumor cells (×200), (c) androgen receptor status of tumor cells (×200), and (d) progesterone receptor status of tumor cells (×200).

A 43-year-old female patient with breast cancer since 2006 has been admitted to our hospital with complaints of weakness in legs. In 2006, she received chemotherapy and radiotherapy. In 2010, after detection of metastasis into liver, she had received chemotherapy again. On her neurological examination, she had paraparesis (left lower extremity: 2/5, right lower extremity: 3/5) and urinary incontinence. On her spinal MRI, we detected an intramedullary and highly gadolinium enhancing lesion with regular borders (Figure 1). We performed a total T12 laminectomy and almost totally excised the lesion microscopically. The lesion was very adherent to neural tissue. On postoperative MRIs, we saw that the inner part of the lesion was emptied, but the parts adherent to neural tissue were still detectable (Figure 2). Postoperatively, patient's paraparesis deteriorated (2/5). Pathological examination of the lesion was coherent with metastasis of breast carcinoma. Histopathological examinationof breast tumor revealed tumor infiltration (Figure 3), cytokeratin expression (Figure 4), also mammaglobin, estrogen, androgen and progesteron receptor status (Figures 5(a), 5(b), 5(c), and 5(d)). The patient has been taken into radiotherapy. On her three-month follow-ups, she did not have any amelioration on her neurological examination.

## 3. Discussion

MISC is a rare but severe condition that can cause neurological deficits and threaten life. Most commonly, it is seen on thoracic area (42%) and then on cervical area (31%) [7]. Patients usually complain from back pain and neurological deficit. Symptoms progress rapidly and paraparesis can occur. Most common symptoms are sensory loss (22.1%), weakness (21%), and pain (21%) [8]. We presented a case of MISC on thoracic area that caused progressing paraparesis. In 2013, Rostami et al. reported in a review article that so far there are 85 cases who have breast malignancy as the primary cancer. The localization is detectable in 52 cases (62%). 45 patients have solitary lesions and 17 of them have cervical (38%), 17 have thoracic (38%), and 11 have lumbar (24%) lesions [8]. The best way of evaluating intramedullary metastasis is MRI which gives information on the nature of the lesion and its relation with adjacent tissues. MRI is also helpful for the planning of the surgery. Lesions that do not have a leptomeningeal connection are resected more easily. Otherwise, radical excision is not possible and only a limited portion of the lesion can be resected [7]. The aim of surgery is the maximum resection. Early surgical intervention is necessary for preventing neurological deterioration and improving the quality of life [9]. Adjuvant radiotherapy should be given especially in cases of partial resection [10]. In the review article, written by Rostami et al. in 2013, the number of MISC cases with the only primary cancer being breast cancer is given as 85. The mean age is 51.1 and the mean duration between the diagnosis of breast cancer and MISC is 50.4 months. Median survey after MISC diagnosis is calculated as 6.1 months [8].

## 4. Conclusion

Spinal intramedullary metastasis of breast cancer is an extremely rare situation, but it has a high morbidity and mortality rate. Despite controversies on the best treatment method, maximal lesion resection through microsurgery is necessary for preservation or amelioration of neurological state and increased life expectancy and quality.
